# Consultations for Poland Syndrome: The Essentials for a Thoracic Surgeon

**DOI:** 10.3390/medicina60071178

**Published:** 2024-07-20

**Authors:** Małgorzata Edyta Wojtyś, Dawid Kordykiewicz, Janusz Wójcik, Periklis Tomos, Konstantinos Kostopanagiotou

**Affiliations:** 1 Department of Thoracic Surgery and Transplantation, Pomeranian Medical University in Szczecin, Alfreda Sokołowskiego 11, 70-891 Szczecin, Poland; 2 Department of Thoracic Surgery, “Attikon” University Hospital of Athens, 12462 Athens, Greece

**Keywords:** chest reconstruction, congenital pectoral aplasia, pectoral deformity

## Abstract

Poland syndrome (PS) is a rare congenital musculoskeletal entity occurring in approximately 1 in 30,000 newborns that manifests with variable symbrachydactyly, ipsilateral costochondral deformities, an absence of pectoral muscles, and breast underdevelopment. These have potential impacts on social, somatic, and psychological functionality, often leading affected individuals to seek expert opinions on corrective surgery. Due to phenotypic variability, strict management guidelines are lacking, with treatment decisions often based on the specialist’s personal experience rather than published evidence. Comprehensive imaging with CT and MRI with 3D reconstruction is crucial for providing a descriptive assessment of musculoskeletal defects. Management is multidisciplinary, involving thoracic, plastic, and pediatric surgeons and hand surgery specialists, as well as psychologists and developmental growth specialists. Surgery should achieve both structural and cosmetic correction to reverse the psychological and social impact and achieve patient satisfaction. We aim to provide thoracic surgeons the essential answers for sharing with affected adult individuals during consultations focusing on chest surgical correction.

## 1. Introduction

Chest reconstructive procedures represent an important workload for modern thoracic surgeons, validating their experience and level of skill. A variety of prosthetic materials are combined with old flap techniques to restore defects in shape, strength, and function, as required. Whether it is a minor or major procedure, benign or malignant pathology, traumatic or congenital defect, all are managed as long as there is an indication. In this context, thoracic surgeons must rely on their experience and available literature when consulting individuals who seek expert opinion for difficult, rare, or special cases. Such an entity is Poland syndrome (PS), which historically was observed and described by various authors in the 19th century [1,2]. It is a real rarity, with a reported incidence between 1 in 17,000 and 1 in 100,000 births [3]. The true incidence is underestimated, as it is not recorded in medical records and escapes diagnosis in most of the world. Generally considered a non-genetic disease, it has a low recurrence rate in the same family and an unspecified pattern of transmission. Males are affected three times more than females, and the right side is twice as commonly affected as the left side. A disruption of the arterial supply (subclavian artery or branches) to the affected side around the sixth week of gestation seems to play a key role [4]. A situation in which growing ribs misconfigure the subclavian artery is termed a subclavian artery supply disruption sequence (SASDS) and provides a good ontogenetic explanation. The degree of arterial obstruction and the location of the vessel involvement directs the severity. The more proximal the occlusion, the more severe the presentation [5]. We can assume that individuals seek medical opinion in the most dysmorphic presentations. Although its historical definition is based on upper extremity (symbrachydactyly) and ipsilateral hemithorax deformity (pectoral aplasia, rib deformities), there are many variants. Muscle aplasia or hypoplasia of the pectoralis major or minor, sternocostal muscle head, serratus anterior, latissimus dorsi, and external oblique are common. The mammary gland may be absent, displaced, or hypoplastic with nipple reposition. This is of particular importance in females. The osseous thorax may be normal or depressed as in the pectus due to deformed ribs. The ribs and cartilage can be hypoplastic or with completely absent parts. The most commonly involved ribs are the third to fifth. The sternal ends of aplastic ribs may be fused together, and the sternum may have rotated, as in pectus deformity. There are individuals with bilateral presentations as well as milder presentations or without hand involvement [6]. It must be mentioned that not all dysplastic thoracic deformities are PS. In reality, affected individuals with any degree of ipsilateral extremity and chest deformity are usually referred to the thoracic surgeon with a PS diagnosis in view of possible surgery. A consultation may be the request of an affected individual and their family or a formal referral by another clinician. It can also involve an adolescent or even a child. A variable volume and quality of information for their condition is already known to them, mainly from web searches or previous physicians. It is safe to assume that the discussion will be around correction options of the chest dysmorphia and functional improvement. This is where we must focus. All imaging diagnostic results must be available to explain them to patients and family. Images with 3D reconstruction facilitate the explanation.

Managing PS is complex and requires a holistic, patient-centered approach to develop effective treatment plans, ultimately enhancing the quality of life for those impacted. In this study, we provide thoracic surgeons with the essential answers, focusing on the surgical correction option and based on the available literature on the issue to simplify the information-sharing process.

## 2. Materials and Methods

### 2.1. Eligibility

The PICOTS framework was used to develop eligibility criteria [P = patient; I = intervention; C = comparator; O = outcome, T = timing/timeframe; S = setting/study design] (Table 1).

### 2.2. Information Sources and Search Strategy

A search was performed in the PubMed and Embase databases (last accessed 20 June 2024). We used the following search terms: Poland syndrome, chest reconstruction, congenital pectoral aplasia, and pectoral deformity. Furthermore, we evaluated citations from included studies and literature reviews.

### 2.3. Selection and Data Collection Process

Titles and abstracts of all primary results from the PubMed and Embase databases were screened. The authors examined the primary articles. Subsequently, the authors performed full-text evaluation. Articles were excluded from further processing if they did not comply with the initial eligibility criteria. Extracted data included study-specific information (year of publication, country of authors), followed by individual patients’ data, such as symptoms, diagnosis, treatment, and follow up.

## 3. Basic Classification

Classification acts as a common language in clinical discussions and is a useful way to explain to patients the type or severity of their condition and to match an indicated treatment. Regarding the upper extremity, there is the Gausewitz et al. classification [7] and Al-Qattan classification [8]. These take into account the presence, length, and form of digits and size of the hand and are used by the plastic and hand surgeons to direct treatment. The thoracic deformities be classified into groups or types by various authors. The early work of Fourcas et al. considers three grades of mild, moderate, and severe forms [9]. Grade I features a hypoplastic pectoral major muscle or mammary tissue and mild asymmetry of the chest and breast. Grade II includes aplasia of the pectoralis major, hypoplasia of other thoracic muscles, and greater asymmetry. Grade III is the severe form, with aplastic thoracic muscles and major rib deformations, including the sternum. In a more simplified grouping, Stylianos et al. describes four grades [10]. This classification varies based on the severity of muscular and skeletal anomalies. In the first level, there is hypoplasia of the pectoralis muscles. The second degree involves the absence of the sternocostal head of the major pectoralis muscle. The third category is the total absence of the major pectoralis muscle or both pectoralis muscles. Finally, the fourth type combines hypoplasia or the absence of the pectoralis muscles with skeletal anomalies of the thoracic bones, such as the sternum or rib cage. The more updated and detailed classification is proposed by Romanini et al. and is used by the AISP (Associazione Italiana Sindrome di Poland), which enhances networking and collaboration between specialists working with PS patients [11]. This is termed TBN (thoracic–breast–nipple-areolar complex) and is based on three graded subgroups:

- The T (thoracic) group has four grades: T1, pectoral major and soft tissue hypo/aplasia; T2, sternal deformity or pectus excavatum plus T1 characteristics; T3, rib aplasia plus T1 characteristics; T4 includes all the previous characteristics and describes a deformed chest with missing ribs and musculature.
- The B (breast) group has two grades: B1 for hypoplasia and B2 for aplasia.
- N (nipple-areolar complex) group has three grades: N1 is hypoplasia and dislocation <2 cm, N2 is hypoplasia and a larger dislocation >2 cm, and N3 is an absent nipple.

The above groups and grades are easy to identify to facilitate the explanation to the patient. All classification findings have to be correlated with the physical examination and imaging findings.

## 4. Diagnostic Imaging

A proper consultation involves a detailed physical examination and is not completed without a detailed review of the imaging studies. As this syndrome may require some form of thoracic intervention, the availability of imaging during the consultation is mandatory. Inevitably, nowadays, the choice is between computed tomography and magnetic resonance. There, we can identify the unilateral absence of pectoralis muscles and other soft tissues, absent or hypoplastic ipsilateral ribs, and sternum and breast abnormalities. A 3D reconstruction image is an excellent presentation aid, especially when there are rib abnormalities. CT provides superior resolution of bone structures, but MRI offers a more detailed assessment of the soft tissues [12]. Cross-sectional imaging with CT using low-dose techniques is better reserved for patients being considered for surgical correction. One thing to take into account is the radiation exposure in younger individuals or females of reproductive age. The MRI is a radiation-free investigation but of higher cost. The popular standard chest radiography is a low-cost, basic imaging investigation that can show rib and sternal irregularities, side asymmetry, and different transradiancy of the affected side due to volume reduction in the overlying soft tissues [13]. Ultrasonography of the chest and upper abdomen was popular three decades ago. It is not an obsolete investigation. It may detect dextrocardia, which is the most described co-existing abnormality, as well as solid visceral organ abnormalities [14,15]. According to Baldelli et al., ultrasound is recommended as the primary diagnostic imaging method to support or confirm the clinical diagnosis of PS due to its easy availability, cost effectiveness, and lack of radiation [16]. Authors suggest that for optimal accuracy during ultrasound examinations, a high-frequency probe set to musculoskeletal parameters should be utilized. The examination should comprehensively evaluate all three heads of the pectoralis major muscle using sagittal and transverse scans, along with the pectoralis minor muscle and the breast tissue. This approach allows for a thorough assessment of agenesis and facilitates appropriate classification of the defect. However, as noted by Romanini et al., distinguishing between the three components of the muscle (clavicular, sternocostal, and abdominal heads) may be challenging for inexperienced doctors [17]. Moreover, imaging has an additional role apart from diagnosis and explanation. It helps the patient to understand the type of work it takes to correct the deformity.

## 5. Conservative or Surgical Treatment

Not all affected individuals have surgery on their mind when walking into the consultation room. The choice for conservative treatment versus minor or major thoracic surgery for PS depends mostly on the defect extent. Indications for corrective surgery may be aesthetic (the most common indications) or functional. Depending on the TBN classification, different reconstruction methods are possible. Functional indications are usually paradoxical breathing caused by significant rib agenesis and compression of the thoracic organs in the presence of pectus excavatum. Typically, chest wall anomalies in PS do not cause significant respiratory distress. The method of reconstructive treatment depends on the type of chest wall anomaly, indications (aesthetic, functional or both), age, development, and psychological assessment [16]. In approximately 25% of patients with PS, these defects are significantly severe; in the remaining ones, they are mild. Indications for the surgical treatment of these defects are similar to those in patients without PS. Attention should be paid to the choice of surgical technique in cases of thoracic asymmetry. Indications relate to the assessment of symptom severity and measurements of chest indicators [18,19]. Open methods are still largely used, but less invasive methods and conservative treatment are increasingly being used, such as a vacuum corset in cases of pectus excavatum or dynamic compression systems, e.g., an FMF system in pectus carinatum. The condition for using conservative methods is a flexible thoracic cage; this is mainly the case in pediatric patients (unossified ribs and flexible sternal attachments) [18]. These methods are described later in this article.

For chest wall reconstruction surgery, general anesthesia is the first choice. Succinylcholine and inhalational agents are contraindicated in patients with PS [20]. In PS patients, a chest wall defect without underlying bone or muscle makes this condition similar to an open chest. The negative pressure generated during inspiration leads to an in-drawing of the chest wall in the region of the defect and vice versa during expiration. Patients with PS can be asymptomatic and have paradoxical breathing. Uncontrolled spontaneous respiration under general anesthesia may worsen paradoxical breathing and cause cardiopulmonary collapse. Therefore, controlled positive pressure ventilation is chosen during the procedure [15]. In addition to the possibility of paradox breath and decreased respiratory muscle strength, the lung at the side of the deformity is more likely hypoplastic or smaller. Therefore, these patients may experience a profound respiratory depression postoperatively [14]. Pain management with regional anesthesia may help to avoid additional respiratory suppression caused by intravenous analgesics. The sternum is innervated by intercostal nerves T1–T6, and thoracic paravertebral nerve blocks with ropivacaine and epinephrine may be helpful. Thoracic epidural anesthesia is used for the Nuss procedure. Alternatively, vertebral anomaly in PS patients may increase the risk of complications of epidural analgesia. A recent study reported that bilateral paravertebral nerve blocks are equal to thoracic epidurals for postoperative pain management in children undergoing the Nuss procedure [19]. A single-shot bilateral thoracic paravertebral nerve block with 0.25% ropivacaine and 1:200,000 epinephrine can benefit patients during the first 24 and even 48 postoperative hours following the Nuss procedure in children [20].

## 6. Age for Surgical Correction

The correct timing for correction is unspecified, but early correction has multiple psychosomatic benefits [21]. Hand deformities must be corrected early so that crucial daily functions, skills, and psychological development are uninhibited [22]. Thoracoplasty in childhood is unnecessary, as respiratory symptoms are uncommon. There is strong published evidence that rib cage defects do not indicate thoracoplasty per se during childhood. Planning for corrective surgery should start during the growth period, even if surgery takes place later in life. Regarding pediatric patients, we believe that pediatric patients should be treated exclusively at children’s hospitals. A chest wall reconstruction is a major procedure with potential complications and a strong impact on breathing physiology. The postoperative demands (i.e., pain management, antibiotic regimens, nutrition, physiotherapy) for children are better met by pediatric healthcare specialists in a dedicated hospital/intensive care unit. A thoracic surgeon may join a team of pediatric surgeons in a children’s hospital and provide their expertise there. Regarding prostheses, we should preferably avoid non-resorbable materials before the age of 12, as advised in the study conducted by Baldelli et al. [16].

## 7. Prosthetic Materials and Flaps Used for Reconstruction

Due to the varying severity of the disease, repair may be conducted in one or more stages. In patients with a milder form of the disease, a single-stage repair is planned optimally after reaching maturity. For children with severe deformities, a two-stage repair is recommended. The initial stage, focusing on the thoracic cage, should begin early in childhood [23]. In adults with fully developed tissues, the primary approach is to ensure the simultaneous correction of both structural and cosmetic deformities. The treatment may combine various methods capable of addressing both the breasts and the chest wall, including breast implants, muscle flaps, autologous fat cells, and other techniques. The initial step for patients with a displaced nipple often involves the implantation of a tissue expander due to the limited amount of subcutaneous tissue. The expansion of this tissue must be conducted very slowly to avoid skin complications. During this period, fat grafting can be performed to reinforce the tissue. The final step is to replace the expander with a breast implant [24]. In other patients, reconstruction can primarily be performed using a prosthetic implant. Furthermore, when planning to reconstruct the chest wall deformity associated with PS, various techniques used for repairing pectus excavatum should be considered. One of the first techniques described in 1949 was presented by Ravitch et al. and involved the excision of all deformed costal cartilages with their perichondrium, the division of the xiphoid and intercostal bundles from the sternum, and a transverse sternal osteotomy to displace the sternum anteriorly [25]. However, a disadvantage of this technique was that it did not address cosmetic reconstruction. Another effective technique introduced by Nuss et al. involves no cartilage resection. This approach is considered particularly suitable for patients with intact chest walls, as they provide support for the bar while it applies pressure to the sternum [26]. Fonkalsrud et al. also introduced a modification to the Ravitch et al. procedure. This technique involves a small incision and selectively removes damaged cartilage segments, preserving the perichondrium to facilitate cartilage regeneration [27]. The subsequent steps focus on the cosmetic repair of the affected tissues. To replace the removed pectoral muscle, composite myocutaneous and simple latissimus dorsi flaps are introduced, becoming common reconstructive procedures after radical mastectomy [28]. When combined with silicone implants, this approach has become a standard complement to the Ravitch method, ensuring an aesthetic outcome [29]. Urschell et al. described this method as follows: chest wall defects are repaired with Marlex mesh. The breast prosthesis is placed directly on the mesh, and a myocutaneous flap of the latissimus dorsi is transferred to cover the prosthesis. A pectus carinatum excavation or defect, if present, is repaired on the side opposite the breast augmentation and myocutaneous flap [30]. Rib defects can be addressed using either absorbable or non-absorbable meshes. Absorbable meshes may play a role in pediatric patients whose thoracic structures have not yet fully matured. Conversely, although resorbable prostheses offer certain benefits, they are temporary solutions, and their application is often postponed until the patient attains a specific age.

One of the most frequently used methods for treating patients with PS is to supplement tissue deficiencies with implants made of various materials. This approach is primarily aimed at improving aesthetic outcomes. This procedure is used in patients with mild chest deformities. It is generally considered safe, but it carries the risk of long-term consequences, such as capsular contracture and the need to replace the implant over time [31]. Breast reconstruction in women commonly involves the use of silicone implants, which are crucial for achieving the desired cosmetic appearance and ensuring patients’ mental comfort [32]. However, frequently, achieving an aesthetically satisfactory outcome requires complementing this method with lipofilling or breast reduction surgery on the healthy side. Options for aesthetic breast reconstruction encompass expanders, implants, and the transposition of the latissimus dorsi flap. Its proximity to the chest wall and breast allows for a pedicled ipsilateral flap reconstruction, providing a significant advantage. Its size is typically sufficient to replace the absent pectoralis major and minor muscles. However, as the authors note, the latissimus dorsi flap, once considered the gold standard in breast reconstruction, is increasingly being replaced by alternative methods, especially in patients with a low BMI. Importantly, in these patients, decreased shoulder strength and functional limitations are observed, along with secondary atrophy of the flaps and poor cosmetic outcomes. Additionally, the latissimus dorsi muscle is often not significantly well-developed in patients with PS [24,33]. Lantzsch et al. suggested that in the case of an ipsilateral latissimus dorsi muscle defect, reconstructing the chest wall with a microsurgical or pedicled transfer of the rectus abdominis muscle (TRAM) might be the best choice. If the axillary vascular system is defective, the internal mammary vessels are used as recipients for free TRAM reconstruction [34]. Another method, considered by some to be a second choice, is the transverse myocutaneous gracilis (TMG) flap, primarily due to its versatility, consistent and reliable anatomy, and relatively simple flap harvest, despite also having the disadvantages of a relatively small flap size and the risk of muscle atrophy [35]. Several papers have highlighted advantages such as low donor site morbidity and complication rates, as well as high patient satisfaction [36,37]. A frequently described alternative procedure is the use of the deep inferior epigastric perforator (DIEP) flap. This method is characterized by a relatively large size, favorable scar location, and stable results. However, the lack of sufficient abdominal tissue in slim patients can limit the use of the DIEP flap; therefore, it remains a unique option for reconstructing a large breast in patients with a higher BMI [35,38]. While these techniques are generally effective, achieving optimal aesthetic outcomes in the reconstruction of the anterior axillary pillar and subclavian fossa remains challenging [39]. However, in numerous cases, reconstructing the prominent anterior axillary fold may not be necessary. The literature supports that in women with a higher BMI, tissue expansion followed by implant placement may be a sufficient procedure [35,40].

Another effective option for correction is reconstruction with a methyl methacrylate prosthesis. Combining two layers of mesh with methyl methacrylate is considered one of the most popular techniques for shaping the chest wall due to ensuring adequate stiffness and protection of the structure [41]. This method is particularly recommended for large anterior chest wall defects, as it allows for individual remodeling of the defect based on the shape of the patient’s chest. It prevents paradoxical movement and achieves satisfactory aesthetic results [42]. However, this method also has its drawbacks: technical difficulties, a long time required for surgery, and perioperative complications, including infection, for instance [43].

Titanium bars combined with expanded polytetrafluoroethylene mesh as modern rigid implants for the reconstruction of large anterolateral defects or large posterior chest wall defects are established in use by modern thoracic surgeons [43]. In one of the articles, the authors described that for patients who have reached skeletal maturity, reconstruction of the thoracic cage with a customized titanium implant should be considered. This approach avoids the need for rib grafts and improves chest wall symmetry [44]. This prosthetic material can also be used in vertical rib osteosynthesis systems such as the vertical expandable prosthetic titanium rib (VEPTR), which serves to stabilize and expand the chest wall, allowing for better lung development and function. Drebov et al. suggested that the VEPTR system is a preferred treatment for young patients with PS to prevent late complications after the child reaches walking age [45].

Autologous fat injection is described as the simplest, fastest, and least complicated technique for correcting breast deformities [46,47,48]. It is now widely used as a stand-alone method in mild cases or as a complement to other procedures. A particular advantage of this method is not only the increase in breast size but also the very satisfying shape and consistency of the breasts. The primary drawback, however, is the inability to achieve the desired effect with a single treatment. Multiple procedures may be necessary to achieve the desired volume [49]. Additionally, as the authors emphasize in one article, lipomodeling does not interfere with breast ultrasound surveillance, allowing for safe and quick diagnostic control [50]. This method is frequently employed to complement silicone implants. In cases where the implant is well tolerated, several sessions of lipomodeling can be added to enhance reconstruction. This may involve increasing the size of any existing pectoral muscle through direct injection or improving the thickness of the skin overlying the implant [51]. However, if the implant is poorly tolerated and the skin is very thin, risking exposure, a conversion from implant reconstruction to autologous reconstruction is recommended [48].

## 8. Discussion

PS is a rare congenital defect combining thoracic and upper-limb deformations on the same side. The defect is usually one-sided [18]. Despite the deformation of the thorax, it rarely results in functional disorders of the organs located there. Mainly due to the rarity of Poland’s anomaly, there are currently no strict guidelines for the management of this syndrome. Italian authors from the IASP have developed a classification system for PS-TBN, which is helpful in planning treatment [13]. Each patient with PS requires an individual and multidisciplinary approach to plan and conduct complex treatment, usually involving a thoracic surgeon and a plastic surgeon, sometimes also an orthopedist or a hand surgeon due to the frequently accompanying upper-limb defects. Anesthetic problems may be important in these patients. Pre- and postoperative rehabilitation procedures are also important. A psychological consultation is also recommended. Indications for thoracic surgery in PS are most often for aesthetic reasons and relatively rarely for functional reasons because, despite the deformation of the chest wall, as already mentioned, respiratory failure or other functional disorders of the chest organs rarely occur. Although the dysmorphological features of PS are quite well defined, the syndrome may have various manifestations and additional defects affecting not only the chest but also defects of the urinary system (renal hypoplasia), neurofibromatosis, microcephaly, and Moebius syndrome. A right-sided position of the heart has also been described [19,52]. A relationship is observed between various syndromes and congenital diseases and an increased incidence of solid malignancies in these populations (e.g., breast, lung, stomach, myosarcoma), as well as Hodgkin’s and non-Hodgkin’s lymphomas [53,54]. The incidence of breast cancer in patients with PS is higher than that in the general population, although not all authors agree with this notion [55,56]. Breast cancer is quite often described in the literature in middle-aged and elderly patients with PS on the side affected by the syndrome. This has implications due to the possibility of breast reconstructive surgery and conservative oncological treatment (chemotherapy, radiotherapy, and hormone therapy are similar). Reconstructive treatment after mastectomy or tissue-sparing surgery may not be possible or may be significantly more difficult. Moreover, when using radiotherapy, the lack of protective function of atrophied muscles for the chest organs may be important [57]. Increased oncological vigilance is recommended in patients with PS, especially breast cancer [58]. These details have to be communicated during the consultation.

Radiological imaging plays a very important role in treatment planning. Currently, CT and MRI with 3D reconstruction are usually used. These tests allow us to confirm clinical suspicions and comprehensively assess any accompanying developmental defects. Due to technical development and the increased availability of the above-mentioned imaging methods, ultrasound has clearly lost its importance [59]. On a chest radiograph, PS is visible as a unilaterally clear lung due to the asymmetry of the chest—sometimes, it is the first diagnosis of PS, and the chest X-ray is taken due to a chest injury or respiratory symptoms, such as cough and shortness of breath [12,60,61].

Italian authors belonging to the IASP propose the following procedure depending on the T and N stages of the TBN classification: In stages T1 and N1, an implant with a fat graft is recommended, possibly in selected patients. At T1 and N2–N3, the use of an expander with or without a fat graft is suggested. In grades T2–T4, thoracoplasty should first be considered if rib aplasia occurs, whether or not it is accompanied by a pectus carinatum or excavatum. When the aplasia covers 1–2 ribs, a Goretex patch can be used, and when at least 2 ribs are affected by aplasia, a metal frame or a custom-made frame using the 3D technique and a Goretex patch can be used. If pectus excavatum is present, a corset or FMF is recommended. In the presence of cobbler’s chest without significant symptoms, a vacuum bell is used, and in the presence of symptoms and specific chest measurement indicators, the Nuss procedure is performed. Only then is the reconstruction of the mammary gland considered [17]. Chest wall reconstructions are large and serious surgeries with the possibility of serious complications and a significant impact on breathing. The choice of a corrective treatment option must not only include an improvement in the appearance of the chest but, above all, take into account functional improvement. There is currently an increasing trend towards the less invasive treatment of chest deformities with PS. For example, currently, fewer large-flap plastic surgery procedures are performed with free or pedicled flaps, in favor of, whenever possible, methods using prostheses or expanders and conservative methods. Surgical methods used in chest deformities and patients with PS can be divided into those that involve reconstructing the contours of the chest wall—the transplantation of muscle flaps (e.g., the latissimus dorsi muscle with endoscopic assistance) and skin-fat flaps of the patient’s own tissues, lipografting, injections of polymer solutions, the use of prostheses, and combinations of these methods—and the reconstruction of the chest bone scaffold—the reconstruction of rib and sternum defects or corrective surgery for lung hernias. In the case of large deformations of the ribs or sternum causing functional or significant aesthetic disturbances, the first step is usually to reconstruct the chest bone scaffold. These include thoracoplasty surgeries such as Ravich’s or Nuss’ surgery with various modifications, also using the patient’s own cartilage or osteochondral grafts or using artificial materials. Ravich’s operation can be performed using a thoracoscope [62,63]. Reconstructive surgeries within the chest wall and the correction of associated defects can also be performed at the same time, which reduces the number of surgeries in a given patient [12].

## 9. Conclusions

The more the body image of young people is exposed and criticized both in real life and on social media, the more affected individuals will request the opinion of medial experts. A dysmorphic chest will certainly lead the affected youth to the consultation office of a thoracic surgeon. The thoracic surgeon has become the real general surgeon of this century, as their expertise spans from trauma and oncology to the reconstruction of rare thoracic deformities. A storm of questions is expected and solid knowledge required to provide scientifically valid answers, especially in view of major corrective surgery. Due to the variability and complexity of Poland syndrome, treatment plans predominantly rely on specialist experience and the existing body of published literature rather than established guidelines. Therefore, in the case of rare diseases, an international, comprehensive, integrative database is essential for specialists across various fields to remain current with the latest requirements and procedures.

## Figures and Tables

**Table 1:** Eligibility criteria for this review based on the PICOTS framework.

	Inclusion Criteria	Exclusion Criteria
Patient	Confirmed presence of Poland syndrome	-
Intervention	Diagnostics, surgical treatment	-
Comparison	Control case or cases not required	-
Outcomes	Final diagnosis, successful treatment	-
Timeframe	Papers published since 1949	-
Study design	Case report or case series	Articles in languages other than English

## Data Availability

Not applicable.
